# Supplementary material for: Are carbon emissions trading and green financial instruments synergistic? -Comprehensive quantitative research based on content analysis
Source: PLoS One. 2024 Mar 7;19(3):e0298601. doi: 10.1371/journal.pone.0298601 (PMC10919601; doi:10.1371/journal.pone.0298601)
Supplement: S1 Data — (ZIP) [file pone.0298601.s001.zip › Data/地级市Z_F工作报告.zip!/┴╔─■/╔≥╤⌠╩╨/└┤╫╘╣1⁄2╓┌║┼í╛┬φ┐╦╩2╛▌═°í┐_2004.docx]

各位代表：

　　现在，我代表市政府向大会作工作报告，请予审议，并请各位政协委员提出意见和建议。

　　一、2003年政府工作回顾

　　过去的一年，在省委、省政府和市委的领导下，在市人大法律监督和市政协民主监督下，我们带领全市人民高举邓小平理论伟大旗帜，认真践行“三个代表”重要思想，经济和社会事业取得了长足的进步。

　　2003年全市国内生产总值实现1602亿元，同比增长14.2％；固定资产投资完成580亿元，增长44.1％；社会消费品零售总额实现776.3亿元，增长11.7％；财政收入达到125.6亿元，增长30％；城市居民人均可支配收入达到8000元，农民人均纯收入达到3818元，同比分别增长13.5％和9.1％。

　　我市一些主要经济指标和增幅，创造了沈阳近10年来的最高水平。固定资产投资、地方财政收入的增幅，列全国15个副省级城市第一位，其它一些主要经济指标增幅也名列前茅。这种情况已多年不见了。由于全市主要经济指标连续3年实现高速增长和协调增长，还由于增速一年比一年加快，我们完全有理由说，沈阳的经济和社会事业发展已经进入快车道，沈阳人民正在意气风发，创造自己城市的新辉煌。

　　（一）“项目年”活动取得丰硕成果，对内对外开放工作登上新台阶

　　去年，市委、市政府决定在全市开展“项目年”活动，经过一年努力，产生了多方面的效应。它不但大大增强了沈阳的经济实力和外向度，同时也大大坚定了全市人民振兴沈阳的勇气和信心。

　　“项目年”促进了对外开放，全市直接利用外资完成22.4亿美元，同比增长59.2％；实现进出口总额41亿美元，增长43.6％，其中出口实现20亿美元，增长42.8％。全年市领导会见了来自32个国家和地区820批7800人次的宾客，其中包括爱尔兰总统、喀麦隆总统、韩国和新西兰前总理及德国宝马、美国通用电气等23家世界知名企业的代表；新开通和恢复了沈阳至福冈、新加坡、釜山等5条国际航线，使沈阳的国际航线达到19条，成为东北地区开通国际航线最多的城市；“韩国周”、第二届“制博会”等大型国际性活动的成功举办，使沈阳的国际知名度和影响力进一步提升。

　　“项目年”促进了对内开放，全市引进规模以上内资项目1272个，总投资额1030亿元，调入资金370亿元，其中超过亿元以上的大项目180个。

　　“项目年”促进了民营经济的发展，“项目年”在引进民营企业的同时，也在加速本地民营经济的发展。目前全市个私经济单位已达23.8万户，从业人员126万人，民营经济实现增加值864.1亿元，同比增长16.5％，占全市国内生产总值的54％。

　　“项目年”促进了固定资产投资的增长，全市固定资产投资增幅超过44％，为历史罕见，为沈阳未来发展增添了后劲。

　　“项目年”促进了政府机关作风的转变，各级领导和机关干部的精力高度凝聚在项目上，这不仅推进了干部自身素质的提高，也提高了机关办事效率，还形成了抓实事、出实效的风气。

　　（二）全面贯彻工业立市方针，结构调整迈出新步伐

　　根据党的十六届三中全会精神，在深入调研论证的基础上，市委、市政府编制了《沈阳老工业基地调整改造振兴规划》，确定了到2010年的发展战略和阶段性工作目标。

　　围绕所有制结构调整，我们实施了国有大企业整体合资，中小企业全部放开，问题企业破产重组。去年有8户大型国有企业完成了整体合资，414户国有中小企业基本完成了转制，22户资不抵债企业实施了破产。各级财政支付8.5亿元，完成了14万名国有企业下岗职工向失业保险并轨。

　　去年规模以上工业实现增加值300.4亿元，同比增长33.5％；规模以上工业总产值实现1252亿元，同比增长33.3％。工业对经济增长的贡献率高达63.9％。汽车及零部件、装备制造和电子信息三个主导产业增加值占规模以上工业的53％。去年第二产业增加值占全市 G DP的47.4％，是90年代以来的最高水平，也是近10年来第二产业增加值首次超过第三产业，居三次产业的第一位，这些都是贯彻工业立市方针和调整经济结构的重大成果。

　　在前年将铁西区和沈阳经济技术开发区合署办公的基础上，去年又将铁西新区面积拓展到126平方公里。通过搬迁、合资、转制等方式解决了一批国有老企业的问题；新上工业项目534个，新增工业企业261家，工业总产值增长32％；财政收入和固定资产投资同比分别增长66.3％和100.4％；城区面貌发生了令人瞩目的变化，铁西新区呈现出大调整、大发展的强劲势头和勃勃生机，为老工业基地振兴闯出了一条新路。

　　浑南新区经济发展态势良好。引进并开工建设67个高新技术项目，比去年同期增长50％，总投资额达197亿元，同比提高近一倍。

　　（三）县区经济结构调整成效显著，农村经济全面发展

　　发展畜牧业、退耕还林、花卉果蔬生产和粮食生产是我市去年推进县区经济结构调整、增加农民收入的重大举措。全年新建各类畜牧养殖小区287个，规模饲养畜禽数量已占饲养总量的70％以上；农村植树造林完成130万亩，同比增长140％，是“九五”时期全市造林总和的2.4倍；全市花卉果蔬生产面积达到195.1万亩，同比增长11.1％；粮食总产量达56.6亿斤。农村二、三产业比重已占87.3％，粮、经作物比例稳定在55：45。

　　农业高新技术开发区经过短短一年的发展，初步形成了以乳品制造、肉禽深加工为主导的六大产业。现已入驻企业54户，总投资额48.1亿元，成为农业产业化龙头企业聚集地。

　　举办了一系列县区经济招商会，取得了很大成果。其中新民林浆纸联合生产企业、法库陶瓷城、康平电厂和辽中铸造城等重大项目，将成为四个县（市）经济发展的重要转折点。

　　重新划分了县区财政收入范围和体制基数，提高了县区的财政调控能力和自我发展的积极性。村村通油路、电费同网同价、农村税费改革三件事，给广大农民带来了实实在在的利益。为四个县城和两个副城各补助1000万元，县城和副城建设出现了新局面。

　　（四）商贸服务业日益繁荣，中心城市功能不断拓展

　　去年城乡市场购销两旺，商业区街改造建设全面启动，商业固定资产投资总额达60亿元以上，全市大型超市已发展到24家，连锁店铺总数已达3000个。

　　经过国内外专家的充分论证，借鉴国内外发达城市沿中轴线发展的经验，我们规划并着手开发了集金融贸易、商务办公、文化娱乐等功能为一体的中央都市走廊工程，简称“南北金廊”。首期4块地已陆续开工，加上以前开工的项目总数已达28个。金廊开发建设为国内外客商提供了新的投资空间，提高了沈阳的城市档次，它将承载沈阳未来的大发展。经过3到5年的努力，“南北金廊”必将以全新的面貌展现在世人面前。

　　我们成功地承办了“全国秋季糖酒交易会”等重要活动，形成了对城市经济的有效拉动。全市会展业实现成交额442亿元，同比增长21.8％。春、秋两届“房交会”房屋成交面积349.2万平方米，交易金额81.3亿元，同比分别增长24.9％和33.6％。沈阳成为国内房地产市场最活跃、最规范、最有潜力的城市之一。

　　金融机构存、贷款余额分别达到2654亿元和2183亿元，同比分别增长16.7％和18.1％。全市发放个人消费信贷实现250亿元，同比增长47％。沈阳商业银行在前年已不亏损情况下，去年又大幅度增盈。

　　（五）城市规划拉开新格局，城市基础设施改造取得重大进展

　　去年我们对沈阳城市发展空间重新进行了规划布局。提出了“南北金廊”中轴线和东西浑河中轴线的概念，从而构筑起城市未来发展的大“十”字型基础框架和空间格局。在这个大“十”字周围，有铁西新区、浑南新区、农业高新区、旅游开发区、金融商贸区和一批县区工业园区。可以说，沈阳大发展的格局已经拉开，沈阳大发展的空间已经形成。

　　去年我们继续系统推进了城市基础设施改造，并取得重大进展。

　　在道路整修上，全市共整修284条道路，面积达717万平方米，其中实施了覆盖111条二级以下街路的“小巷工程”，面积为71.6万平方米。全市道路完好率保持在90％以上。特别是东北大马路、浑河大街、沈阳至新民公路等道路的拓宽改造，使我市交通水平和通行能力又有了进一步的提高。

　　在绿化上，新植树428万株，新增绿化面积18.26平方公里，城市绿化覆盖率达33％，人均公共绿地已达8平方米，初步形成了城外森林环抱、城内绿化成网的格局。

　　在水系建设上，建成了长5公里的沈水湾公园，与前年建成的上游五里河公园相接，形成了绵延10公里的沿河绿带和亲水休闲区。对严重污染几十年的8.9公里细河城区段进行了全面清淤整治，并对卫工明渠进行了人工湖配套建设。

　　在大气治理上，拆除烟囱1113根、锅炉房403座，加大对机动车尾气的治理和对扬尘的覆盖，使我市环境空气质量优良天数由前年的203天，提高到去年的298天。

　　在污水处理上，建设了满堂河、沈水湾、仙女河三个污水处理厂，形成了新的污水处理系统，全市污水处理能力由前年的48％提高到去年的70.4％。

　　在垃圾处理上，建成南部老虎冲和北部大辛垃圾处理场，日处理垃圾达到4400余吨，使全市生活垃圾得到100％的无害化处理，结束了沈阳没有垃圾处理场的历史。对二环和三环区域内的环境卫生实行城区化管理，解决了垃圾围城问题。

　　在城市亮化上，除了对10条景观路、8个大型广场的灯饰亮化外，还对592座高层建筑、16块大型绿地和19个公园进行了高标准亮化，使沈阳的城市亮化总体水平达到70％。

　　去年城市基础设施建设投入资金70亿元，其中，市本级投入24.2亿元（包括土地出让金20亿元），区、县及开发区投入29.5亿元，政府部门和企业自筹15.2亿元，招商引资1.1亿元。近三年市本级不仅没有靠贷款搞城建，而且还偿还了2000年以前的贷款11.75亿元。

　　作为老工业基地的沈阳，过去曾被视为脏乱差的城市，经过几年来全市人民的共同奋斗，生态环境和人居环境都发生了令人瞩目的变化。天蓝了、树绿了、水清了、路宽了，广大市民沉浸在城市变化的欣喜中，沈阳人为拥有这样的城市而感到自豪。

　　（六）社保体系逐步完善，就业和再就业工作稳步推进

　　全市参加养老保险的从业人员增加到129.7万人，征收养老保险费超过25亿元，确保了64.5万名退休人员全部按时足额领取养老保险金；参加医疗保险的人员增加到152万人；127.5万人参加失业保险，17.2万人领取失业保险金。最低生活保障制度基本完善，全市有17.3万人享受低保。

　　全年开发就业岗位12.2万个，实现就业和再就业21.4万人次，开发公益性岗位3.7万个，安置大龄下岗失业人员3.7万人。城镇登记失业率为6％。

　　市区两级财政共投入6083万元，用于改造社区办公用房、社区干部补贴和办公经费。目前大部分社区办公用房达到80平方米，社区工作者的月补贴标准已达545元，今年还将继续提高，最终达到全市平均收入水平。

　　经过几年来的不懈努力，在党中央、国务院和省委、省政府的领导和支持下，在沈阳这样一个历史欠帐严重的老工业城市，能够初步建立起比较完善的社保体系，实属来之不易，也是过去难以想象的。目前，我市社会保障工作已经出现转折点，这就是社保体系基本形成并正常运转，社保资金出现结余，再就业已经度过了最困难时期。这对沈阳人来说，不能不说是个历史性的重大成就。

　　（七）科教文卫蓬勃发展，各项社会事业全面进步

　　全年实现高新技术产值756.8亿元，同比增长超过22％。一批处于世界领先水平的项目落户沈阳，有力地促进了我市的产业升级。成功承办了第五届中国科协学术年会。

　　围绕义务教育的均衡发展，着力解决广大市民非常关注的“择校热”和乱收费问题。在和平、东陵两区实施了基础教育综合改革试点，对22所薄弱学校进行改造，校长、教师的交流机制正在形成。实施了以县为主的农村义务教育管理体制改革。去年我市有12所学校被确定为省示范高中。

　　全年引进、培养各类人才3.3万名，引进外国专家1000人次。

　　成功举办了第五届沈阳艺术节、首届新春庙会、元宵灯会等系列活动。全年举办文化广场活动2000余场，活跃了群众的文化生活。

　　近两年投入4亿元资金，对“一宫两陵”实施了建国以来规模最大的内部修缮和外部环境清理工程。申报世界文化遗产的结果将在今年七月份揭晓。

　　在第五届全国城市运动会上，我市代表队取得了好成绩。在全市128个社区安装了健身器材，全面推进了群众体育活动的开展。

　　公共卫生服务体系建设进一步加强，农村卫生医疗条件进一步改善，城市两级医疗服务格局初步形成。

　　面对突如其来的“非典”疫情，全市上下总动员，夺取了非典病例和疑似病例“双零”的重大胜利。对此，国内外朋友给予了高度的评价和赞赏。这一重大胜利，充分展示了720万沈阳人民精诚团结、奋力拼搏、共渡难关的伟大精神，同时也显示了沈阳城市的管理水平。这进一步树立了沈阳在世界的形象，进一步激发了沈阳人民昂扬的奋斗精神。

　　（八）依法治市工作继续推进，市民生活不断改善

　　去年经市人大通过、省人大批准，出台了《沈阳市城市道路管理条例》等6件地方性法规，市政府制定了《沈阳市城市垃圾管理规定》等10件政府规章。开展了行政执法大检查、重大行政处罚决定的备案和行政执法投诉的案件受理工作。加大了对“法轮功”等邪教组织的打击力度，成功侦破了“1?18”持枪爆炸抢劫运钞车等重大案件，治安形势进一步好转。全市上下认真做好群众来信来访工作，维护了社会的稳定。各级政府认真接受人大依法监督和政协民主监督，市政府定期向市人大主任会议和市政协常委会议通报政府工作情况。全年办理人大代表建议1070件，办理政协提案736件，办复率分别达到100％。

　　加大了党风廉政教育和反腐败的力度，在政府处级以上干部中开展了专项述廉活动。

　　行政审批制度改革取得成效，办事大厅运转良好。

　　去年我市被评为“全国双拥模范城”，已连续5年获此殊荣。

　　去年初确定的为城乡群众办好35件实事已全面完成。其中重点有：为低保户和困难企业补贴采暖费1.2亿元；棚户区改造拆迁3.6万户；新增公交车1000台；开发扶贫项目814个，覆盖258个村，受益农民达2.4万户；筹集资金500万元，保证了2000余名贫困家庭大学生顺利入学等。此外，我们还采取由政府资助和社会广泛募集资金的办法，向2.2万户没有电视机的农村贫困家庭捐赠彩电。

　　计划生育、统计、审计、物价、参事、民族、宗教、广播、电视、新闻、出版、侨务、质量技术监督、药品监督、安全生产、民兵、预备役、人防、档案、修志、文史、残联和老龄工作也都取得了许多新成果。

　　回顾过去的一年，我们的工作之所以取得显著的成效，是市委正确领导的结果，是市人大和市政协监督和支持的结果，是全市各条战线同志们勤奋工作的结果，同时也是国内外朋友热心帮助的结果。

　　在这里，我代表市政府，向投身于沈阳改革开放和现代化建设的各族人民致以亲切的问候和崇高的敬意！向给予我们支持与监督的人民代表、政协委员，各民主党派、工商联和无党派人士、人民团体，向参与沈阳发展建设的中央和省在沈单位、驻沈部队指战员、武警官兵和在沈工作的外籍人士、港澳台同胞、海外侨胞及国际友人表示衷心的感谢！

　　总结一年来的工作，我们也清醒地认识到，当前还面临着一些亟待解决的矛盾和问题。由于旧体制的长期影响，沈阳的市场化程度仍然较低；城市的国际化水平和经济外向度还不高；具有牵动力、百亿元以上的大型工业项目仍然缺少；国有企业改革的战略任务还远没有完成；政府职能的转变仍然存在较明显差距；作为区域性中心城市，沈阳仍然缺乏对周边地区经济发展应有的拉动和辐射作用；群众生活方面还有许多难点问题没有解决，社会还存在许多不稳定因素。上述这些问题，有待于我们在今后的工作中认真加以解决。二、2004年政府工作安排

　　根据党的十六届三中全会和中央11号文件精神，市委十届八次全体会议提出了振兴沈阳老工业基地的总体目标，这就是要经过七年的不懈努力，完成沈阳老工业基地调整改造的主要任务。集中做强汽车及零部件、装备制造、电子信息、化工医药、农产品加工等五大产业；加快构筑新型工业、先进文化、模范生态、法治诚信“四位一体”城市；努力形成全国装备制造、东北地区商贸物流和金融三大中心，成为辽宁乃至东北地区全面振兴的重要增长极。

　　届时，沈阳的经济总量将大大增加；届时，沈阳对东北地区的辐射作用将越来越大；届时，沈阳这座城市的面貌将发生巨大的变化。我们要承担起“东北大振兴，沈阳要先行”这一时代赋予我们的光荣使命，去干一番轰轰烈烈的伟大事业。

　　2004年政府工作的指导思想是：认真贯彻党的十六届三中全会精神，紧紧抓住振兴东北老工业基地的战略机遇，坚持以人为本，以改革开放为动力，以抓重点项目为中心，以开展“环境年”活动为切入点，继续改善经济增长方式，促进经济快速增长，加强城市公共设施建设，大力提高城市综合管理水平和市民素质，着力扩大就业和再就业，做强做大县域经济，努力提高城乡人民收入水平，积极拓展中心城市功能，实现经济、社会和环境的全面协调和可持续发展。

　　今年全市经济和社会发展的主要预期指标是：国内生产总值增长12.5％，财政收入增长14％，固定资产投资增长25％，直接利用外资增长10％，社会消费品零售总额增长11％，城市居民人均可支配收入和农民人均纯收入分别增长10％和8％。

　　（一）狠抓结构调整，促进工业经济快速增长

　　振兴老工业基地的核心是结构调整。今年要基本完成国有大型企业股份制改造的任务；全面完成中小企业的规范转制和收尾工作；50户问题企业实施破产退出。在国家政策支持下，努力解决沈阳国有企业人员和债务两大难题。逐步完成30户中省直工业企业的主辅分离任务。

　　铁西新区要继续发挥老工业基地振兴中的先行作用。要规划建设100个汽车零部件项目和100个装备制造业项目，固定资产投资争取超过100亿元。

　　浑南新区要加速带动全市工业的优化升级。要做大三宝、 L G产业链规模，加快东软数字医疗器械等项目的实施进程。全区技工贸总收入、工业增加值、固定资产投资都要力争增长30％以上。

　　要把发展民营经济放到更加突出的战略地位。没有民营经济的大发展，就没有经济结构的大调整。要以更大的工作热情，扶持本地民营企业发展，同时要吸引1500户外地民营企业进入沈阳，积极鼓励民营企业参与国有企业的战略性重组，努力使我市民营经济的增幅达到20％以上。

　　经济结构调整的最终成果，是要形成一批具有强大牵动力的大型骨干企业。为此，近几年我们必须着力培育出10个超百亿元的大型工业企业。而要实现这个目标，就必须抓项目，抓项目仍然是我们今年经济工作的重点，这项工作的力度不仅不能减，还要继续加大。

　　（二）积极调整农村经济结构，大力发展县区经济

　　以县区财政和农民收入增长为重点，积极发展县域经济，认真解决“三农”问题。在保证粮食生产的基础上，面向国内外市场，大力发展畜产品、花卉、蔬菜、水产品和林果业，加快农业产业结构调整的步伐。发挥林业资源优势，抓好林产品深加工；畜牧业规模化养殖比重达到80％，畜牧业产值力争达到85亿元；花卉种植面积力争达到3万亩；新增蔬菜种植面积19万亩，要围绕农副产品深加工，上一批大项目。

　　今年要在重点抓好业已确定的县区重大项目的同时，还要引进一批投资1000万元以上的项目。

　　深入抓好农村税费改革，努力化解村级债务，严格土地管理，妥善解决土地征用过程中保护基本农田和农民补偿问题，维护农民的合法权益。加强农业水利基础设施建设，提高抗灾水平和能力。农村劳务输出要达到10万人。

　　继续给予县城和副城各1000万元的资金支持，进一步搞好小城镇建设。

　　农业高新区要加速发展，全区工业总产值、工业增加值、固定资产投资、财政收入都要以50％以上的速度增长。年内要引进3000万元以上的项目40个。

　　（三）进一步扩大对外开放领域，不断提高城市外向度

　　今年，我们要继续把承接韩日产业转移和吸引世界500强企业作为对外招商的重点，年内力争再引进10家世界500强企业在沈阳落户；促进一批外国企业在沈阳设立分支机构；在沈阳设立外资银行分支机构3家以上。

　　继续实施海外留学人员回归创业工程，要吸引200名海外高层次留学人员来沈服务，鼓励海外学子创办留学人员企业。

　　继续办好“制博会”、“韩国周”、“民交会”和“海外学子创业周”等大型招商引资活动；积极争办在国内外有影响力的旅游节和冰雪节；今年要在去年来沈旅游人数2272.7万人次的基础上增长25％。

　　继续扩大友好交往的途径，争取新开通5条国际航线；全年要实现外贸出口22亿美元；积极争取外国元首和政要来沈阳访问。

　　在对外开放的同时，必须狠抓对内开放，要以温州、闽南为重点继续引进民营企业，力求取得新突破。

　　（四）开展“环境年”活动，提高城市整体水平

　　市委、市政府确定今年为“环境年”，目的在于全面加强城市软、硬环境建设，提高城市建设和管理水平，提升城市整体形象。

　　加强城乡硬环境建设。继续推进森林城市建设，完成18.7平方公里的绿化任务，建成区绿化覆盖率力争达到40％。对联合路等9条主干道和东北大马路等7条出口路进行全面整修；完成1500公里的村村通油路建设。对浑河、满堂河继续进行治理，新增水面395.5公顷，三环以内水面积率达3.41％，形成新的环城水系。继续拆除锅炉房400座、烟囱1000根，全年大气优良天数力争达到300天。在农村建设10个环境优美乡镇，对100个村屯进行环境综合整治。抓好低洼地区雨后积水整治，完成21条街路的排水管网整修和60处积水点的改造。此外，还要做好地铁一号线工程开工准备工作。继续开展争创国家卫生城和全国环保模范城市活动。

　　强化城市管理。要进一步健全交通工具的准入制度，并严格执行；发展大公交，今年新增1000台公共汽车，逐渐减少自行车数量；加强交通线路的规划，在交通要道建设一批过街天桥和地下通道；通过强化教育和管理，使行人和车辆都能按章通行；加强交通警察队伍建设，下大力改变沈阳的交通现状，努力达到全国一流水平。进一步取缔马路占道市场；规范广告管理，认真整治小招贴和乱涂乱写等行为；进一步完善环境卫生和除运雪工作体系。对4415万平方米的住宅小区进行综合治理，全市住宅小区物业化率和规范化率要分别达到92％和40％；创建30个生态环保模范小区、36个安静小区和100个环境达标社区。

　　加强城市软环境建设。切实转变政府职能，清理削减政府审批事项，提高办事效率，加强对办事大厅的管理，建设行为规范、运转协调、公开透明、廉洁高效的政务环境。要建立诚信体系，建设信用沈阳，政府部门要进一步强化服务意识，加强行政管理和监督检查，创造公平有序的市场环境。

　　城市软环境建设的重要方面是治安问题。我们要做出规划，加大投入，建好队伍，完善法规，全面建设安全城市，从而使我们的市民有更强的安全感，使国内外客人有更强的安全感。同时，要加强公民道德建设，全面提高市民素质，创造和谐的人文环境。

　　（五）加快发展现代服务业，全面提升中心城市的影响力和辐射力

　　继续推进“南北金廊”建设，在已开工的28个项目基础上，今年还要新开工建设一批项目。

　　借鉴国际国内滨水地区开发经验，适时启动浑河两岸的开发，把浑河两岸开发建设成一个集经济带、文化带、景观带为一体的秀丽壮观的滨水地区。

　　加快太原街、中街、长江街和北市等商业街区的整体改造步伐，发展新兴业态。组织承办好“中国沈阳现代物流展”等商业会展活动。

　　继续促进房地产业健康发展。要根据国家有关法规和外市经验，进一步做好房屋拆迁工作。继续举办好春秋两季“房交会”，进一步活跃房地产市场。

　　努力抓好棋盘山国际风景旅游开发区的热带雨林主题公园等大型旅游项目建设，开工建设从棋盘山至国家森林公园全长10公里的绿色生态路。

　　（六）进一步夯实基础工作，继续完善社会保障体系

　　加强对养老和失业保险费的征缴和监察力度，确保应收尽收。基本养老保险参保人数要达到130万人，基本医疗保险参保人数要达到160万人，努力扩大失业保险覆盖面，进一步完善最低生活保障制度，做到应保尽保。

　　健全市、区、街道、社区四级推进就业工作体系。今年全市要开发就业岗位12.5万个，实现下岗失业人员再就业19万人次。大力开发公益性岗位，积极安置就业困难的大龄下岗失业人员。培训下岗失业人员6万人。城镇登记失业率要控制在6％左右。

　　要进一步加强社区服务体系建设，改善社区办公条件，提高社区工作人员待遇，努力推进退休职工的社会化管理。

　　（七）以体制创新为突破口，促进社会事业全面发展

　　全市高新技术产值要达到890亿元，同比增长17.7％。市属科研院所要在去年改革试点的基础上，完成以股份制为主要形式的产权制度改革。大力培育民营科技企业，办好19家创业孵化器。建立健全风险投资体系，为科技人员领办科技企业创造条件。

　　认真贯彻《中共中央、国务院关于进一步加强人才工作的决定》，注重培养和引进高层次管理人才和高等级技术人才。全年要引进100名博士、1000名硕士和1万名本科毕业生，为老工业基地的改造振兴提供人才支持。

　　继续深入实施教育体制和教师人事制度改革，充分发挥两个教育综合改革实验区的示范作用。实施《农村教育跨越发展计划》和《职业教育振兴计划》，加快装备制造工程学校等重点职业学校的建设。继续支持在沈高校的发展，不断提高办学质量和效益。

　　重点推进新闻出版、广播电视等文化产业的改革和发展。全面完成文化体制综合改革试点任务，积极筹办好“中国沈阳杂技节”等一系列活动，年内要建成儿童活动中心和残疾人康复中心，开工建设市图书馆和档案馆，启动一批雕塑项目，提高城市文化品位。

　　建立并完善

　　公共卫生体系。要逐步推进办医形式多样化和投资主体多元化。搞好新型农村合作医疗试点工作。要继续做好“非典”防治工作。完善卫生监督执法体系，公共卫生监督覆盖率要达到100％。要积极发展残疾人事业，依法维护其合法权益。

　　深化体育改革，促进体育产业发展。继续做好2008年奥运会足球分赛场的筹备工作。继续实施全民健身计划，市区要筹集资金新上一批健身器材，使社区健身器材普及率达到50％。要注重培养高层次体育人才，努力提高竞技体育水平。

　　（八）坚持依法行政，努力为人民群众多办实事

　　各级政府要自觉接受人大的依法监督和政协的民主监督，要认真办理人大代表议案、建议、批评和意见，认真办理政协提案，充分听取各民主党派、工商联、无党派人士和人民团体参政议政的意见。

　　进一步完善各级信访、市民投诉及社区人民调解机制，认真稳妥地处置群体性事件，扎实有效地化解人民内部矛盾。

　　继续深入开展拥军优属、拥政爱民和多领域的军民共建活动。

　　切实开展为群众办实事、送温暖活动。要对100所农村中小学危房进行改造；免收贫困家庭学生高中阶段的学杂费，保证当年考入大学的学生能够如期上学；市、区共筹集2000万元，建立城市居民医疗救助基金，解决困难群体就医难问题；实施打井改水431处，解决我市农村40万人饮水含氟量高问题；继续推进“小巷工程”，对全市320条、总面积230万平方米的二级以下街路实施整修；对市区315条无照明路灯和130条照明度差的街巷路，分别安装和更换灯具；建立食用农产品市场准入制度，保障群众食用农产品消费安全。实施房屋“平改坡”125栋，面积40万平方米。年内准备改造棚户区4万户。

　　进一步帮助困难群体改善居住条件并做好城市拆迁工作。年内要建设40万平方米政府补贴房，每户面积为50平方米左右，每平方米价格低于1800元；市区两级政府筹集2500万元，作为困难户房租补贴；拟投入2700万元，新建廉租周转房1.5万平方米；继续在长白地区建设一批低价位房，供动迁户购买。

　　各位代表，同志们！

　　党中央振兴东北老工业基地的号角已经吹响，沈阳加速发展的风帆已经扬起。在新的一年里，全市人民要借此东风，乘风破浪，同舟共济，夺取新的更大的胜利！
